# Obstacle Circumvention and Motor Daily Dual Task During a Simulation of Street Crossing by Individuals with Parkinson’s Disease

**DOI:** 10.3390/life15060900

**Published:** 2025-05-31

**Authors:** Carolina Favarin Soares, Aline Prieto Silveira-Ciola, Lucas Simieli, Patrícia de Aguiar Yamada, Fábio Augusto Barbieri, Flávia Roberta Faganello-Navega

**Affiliations:** 1 Institute of Biosciences, Graduate Program in Human Development and Technologies, São Paulo State University (UNESP), Rio Claro 13506-900, SP, Brazil; carolfavarin@gmail.com (C.F.S.); alineprietobs@gmail.com (A.P.S.-C.); 2 Graduate Program in Movement Sciences, Human Movement Research Laboratory (MOVI-LAB), Department of Physical Education, School of Sciences, São Paulo State University (UNESP), Bauru 17033-360, SP, Brazil; lucassimieli@hotmail.com (L.S.); fabio.barbieri@unesp.br (F.A.B.); 3 Research Laboratory of Neuromuscular Disorders (LIDEN), Department of Physiotherapy and Occupational Therapy, School of Philosophy and Sciences, São Paulo State University (UNESP), Marília 01049-010, SP, Brazil; patricia.yamada@unesp.br

**Keywords:** Parkinson’s disease, gait, dual task, motor control

## Abstract

Parkinson’s disease (PD) causes attentional deficits and worse dual-task (DT) performance, which increases the risk of being run over. In addition to motor deficits, the decision-making ability and the response to external stimuli are impaired. The aim of this study was to evaluate the spatiotemporal parameters of gait during everyday tasks of individuals with PD, specifically during street crossing simulation, obstacle circumvention, and motor DT. People with PD (PG) and matched controls (CG) were distributed into two groups and were evaluated under six different gait and randomized conditions: without a concomitant task (NW); with obstacle circumvention (OC); and four other conditions under simulation of street crossing (without concomitant task (SC); with obstacle circumvention (SC_OC_); carrying bags (SC_B_); and carrying bags concomitant to obstacle circumvention (SC_OC+B_)). The CG group had greater values for all parameters compared to PG, except for double support time. This study’s results found that individuals with PD took smaller, narrower, slower, and shorter steps when compared to neurologically healthy older people and that there was a change in the spatiotemporal gait parameters of all individuals, except for the step-duration parameter under the most difficult crossing conditions.

## 1. Introduction

Accidents involving older pedestrians occur especially when crossing streets, as factors such as short crossing time, difficulty in performing dual tasks (DTs), decreased efficiency in decision-making, and reduced walking speed and step length directly affect safety during displacement [1,2]. Thus, successfully crossing a public road depends on action perception, i.e., the integration of different auditory, visual, vestibular, and visuospatial stimuli [3], to estimate a safety margin before following an efficient travel path. Such a decision relies on individuals’ previous knowledge, based on the need to adjust speed and perceive time, distance, and approaching vehicles during movement [4].

Fontaine and Gourlet [5] demonstrated that the age group from 72 to 85 years had the most impaired executive performance during street crossing, representing 37% of deaths or serious injuries. In Brazil, pedestrians are the third-largest group of fatal victims in traffic accidents, after motorcyclists and car occupants. Among pedestrians, the greatest number of deaths occurs in people over 60 years of age [6]. However, while some studies analyze the decision-making behavior of older adults regarding the gap between crossing space and vehicle approach speed, few consider tasks that occur simultaneously with street crossing, such as avoiding another person or a hole, carrying groceries or a handbag, or even performing all these tasks together [7].

Although the literature on street crossing in neurologically impaired populations remains limited, Parkinson’s disease (PD) deserves particular attention due to the heterogeneous challenges it presents in mobility and cognitive–motor integration. PD is a progressive neurodegenerative disorder characterized by the loss of dopamine neurons [8], leading to both motor and non-motor symptoms [9]. While the exact pathophysiology of PD is not fully understood, it is thought to involve a combination of factors, including alpha–synuclein aggregation, mitochondrial dysfunction, synaptic transport deficits, and neuroinflammation [9,10]. The resulting dopamine deficiency affects not only motor control, but also cognitive and executive functions [11], which are essential for navigating dynamic environments such as street crossings. Core motor symptoms include bradykinesia, muscular rigidity, resting tremor, and postural instability [12], all of which tend to worsen as the disease progresses, contributing to gait disturbances and increased risk of falls and accidents [13].

Ford et al. [14] analyzed the association between motor function, walking speed, sleep vigilance state, and visual processing speed with the safety of street crossing in individuals with PD. They observed that the strongest predictors for the occurrence of accidents were gait speed and visual processing speed, whereas motor functions did not interfere with task performance. Amaral–Felipe et al. [15] studied gait kinematic parameters during street crossing under normal and reduced time conditions but did not include the performance of concomitant tasks in their analysis. Recent studies reinforce that individuals with PD have significant impairments in gait, especially under DT conditions and in complex environments, showing reductions in gait speed and step length, decreased gait smoothness, and increased risk of falls and accidents [16,17,18].

Due to this, to date, no studies have investigated how the gait of individuals with PD behaves during the performance of everyday DTs within the time limits provided by traffic authorities for street crossing. This study differs from previous works by simulating more realistic urban scenarios, incorporating multiple simultaneous challenges (e.g., obstacle avoidance, load carrying, and limited time), which have not been explored in earlier research.

Individuals with PD are at greater risk of accidents when crossing streets due to impaired voluntary and automatic motor performance, which makes it difficult to adapt to the environment, increases distraction levels, and compromises DT performance [19,20,21]. The difficulty in performing DTs in PD lies in the reduced efficiency of directing adequate attention to the required activities. Thus, situations that demand increased attentional focus overload the cortex, which has limited attentional capacity [22,23]. Furthermore, the inability to divide attention leads to a longer time to complete activities, a greater probability of errors and accidents, and reduced adaptability to different external stimuli [3,21,24].

Therefore, when DTs are transferred to a street-crossing context, actions such as carrying a bag or talking on the phone become more dangerous. Additionally, situations that require increased attention, such as avoiding an obstacle or another person, further increase the risk of accidents, especially in individuals with PD. In such cases, individuals with PD may exhibit changes such as increased step variability, greater postural instability, and altered speed and step length, making them more prone to falls and being run over.

Thus, there is a need for studies that assess the impact of simultaneous everyday motor DTs (such as obstacle avoidance and load carrying) during simulated street crossing in people with PD, using protocols that approximate real-life situations and challenges. This approach may provide more ecologically valid insights and support the development of targeted interventions.

The objective of this study was to evaluate the spatiotemporal gait parameters of individuals with PD during everyday tasks, specifically in a simulated street-crossing scenario involving obstacle avoidance and a motor DT (carrying bags). Two main hypotheses were proposed: (i) due to gait alterations and motor planning deficits characteristic of PD, individuals with the disease were expected to exhibit greater walking impairments (shorter step length, lower speed, and longer double support time) compared to neurologically healthy older adults; and (ii) under more complex gait conditions (street crossing combined with obstacle avoidance and carrying bags), both groups would perform worse, but impairments would be more pronounced in individuals with PD.

## 2. Methods

### 2.1. Participants

Eighteen individuals with Parkinson’s disease (PD group, PG) and 18 neurologically healthy older adults (control group, CG) participated in the study. The sample size was determined using G*Power^®^ 3.1, based on collected data for the variable of step length during walking without a concurrent task in a street-crossing condition, with a power of 0.95 and an effect size (d) of 1.12. The calculation indicated a total of 36 participants, with 18 individuals in each group. Eligibility criteria for the PG included a confirmed medical diagnosis of idiopathic Parkinson’s disease, classification within stages I to III of the Hoehn and Yahr scale [25], and the absence of ongoing pharmacological adjustment. Common eligibility criteria for all participants included: age over 60 years; absence of pain; no history of cognitive, cardiovascular, respiratory, or neurological disorders; and no fractures or severe soft tissue injuries within 6 months prior to the study [26,27].

The GC individuals were recruited through the physical activity program offered for older adults at São Paulo State University (UNESP)–Bauru Campus. The PG individuals were recruited through the “Ativa Parkinson” group sponsored by the same university. This project was approved by the local ethics committee (protocol 2.171.624). All participants signed an informed consent form.

### 2.2. Experimental Design and Clinical Evaluations

The research was conducted on a single day at the Human Movement Research Laboratory (MOVI-LAB)–Department of Physical Education, UNESP/Bauru Campus, Brazil. All participants were instructed about the stages of data collection, which included anthropometric, clinical, and motor assessments. The Mini-Mental State Examination (MMSE) [28] was used to assess cognitive function, and older adults were scored according to their education level to determine eligibility for inclusion in the study. The history of falls over the past year was also investigated. The motor condition was assessed only in members of the PD group using the Unified Parkinson’s Disease Rating Scale (UPDRS–Part III), and all procedures were conducted during the “on” phase of PD medication, approximately 1 h after the intake of dopaminergic medication [29].

### 2.3. Gait Assessment Under Different Street-Crossing Conditions

**Figure 1:**
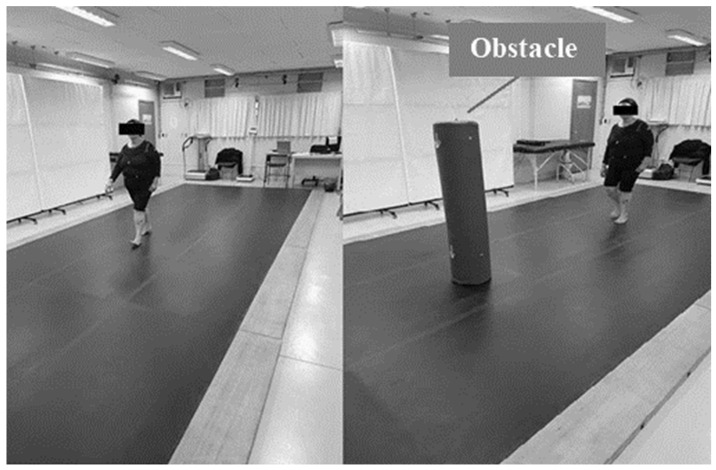
Data collection environment under walking conditions without crossing the street, with and without obstacle circumvention (NW and OC).

**Figure 2:**
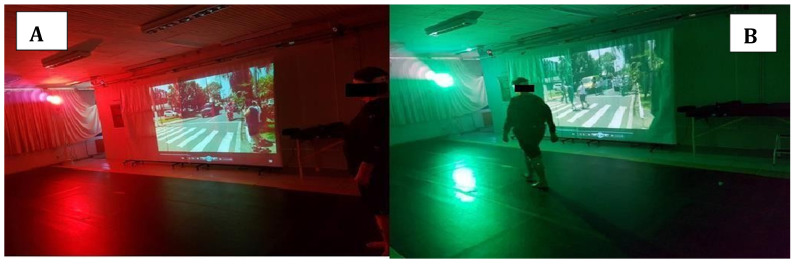
Data collection environment in the walking condition with the street crossing simulator: (**A**) represents the crossing simulation with a red light (closed), and (**B**) the crossing simulation with a green light (opened).

Gait was assessed under six different randomized conditions. In conditions without street crossing (Figure 1), participants were instructed to walk at their usual speed. In conditions involving a street-crossing simulation (Figure 2), participants were instructed to adapt their gait to the duration of the “open” traffic light (8 seconds), indicated by a green light. Condition I (NW): normal walking without street crossing or any concomitant task. Condition II (OC): walking without street crossing, with obstacle circumvention. Condition III (SC): walking with street crossing, without performing any concomitant task. Condition IV (SC_OC_): walking with street crossing and obstacle circumvention. Condition V (SC_B_): walking with street crossing while carrying loads corresponding to 10% of body weight in bags, similar to shopping bags, with the weight equally distributed between both upper limbs. Finally, condition VI (SC_OC+B_): walking with street crossing, carrying bags, and circumventing an obstacle. Each participant walked along a walkway five times under each condition, with the sequence randomized by one of the evaluators. Before starting the gait assessments, participants were familiarized with all conditions to prevent learning effects from influencing the results [30].

For data collection in the non-crossing conditions, participants were instructed to walk at their usual speed. Under all gait conditions, participants began walking when the traffic light turned green (8-second duration). In the street-crossing simulation conditions, participants were instructed to pay attention to a projector (positioned on the right side), which simulated traffic sounds and visual scenes. Based on the video and the pedestrian traffic light simulator, participants decided the optimal moment to cross the walkway, respecting the pedestrian signal duration.

In all conditions, participants were required to walk a straight distance of 8 m along a 3 m-wide walkway. If a participant was unable to complete the task within the traffic light interval, the attempt was excluded from the analysis. In conditions involving obstacle circumvention, participants were instructed to avoid contact with the obstacle and return to the original straight path after passing it. The obstacle was cylindrical (0.35 m in diameter, 1.30 m in height), placed in the middle of the walkway (4 m from the starting point), leaving 1.30 m of clearance on each side [31]. Participants were not instructed on which side to pass the obstacle, only to avoid contact, while also walking with shopping bags in street-crossing conditions.

### 2.4. Data Processing and Analysis

**Figure 3:**
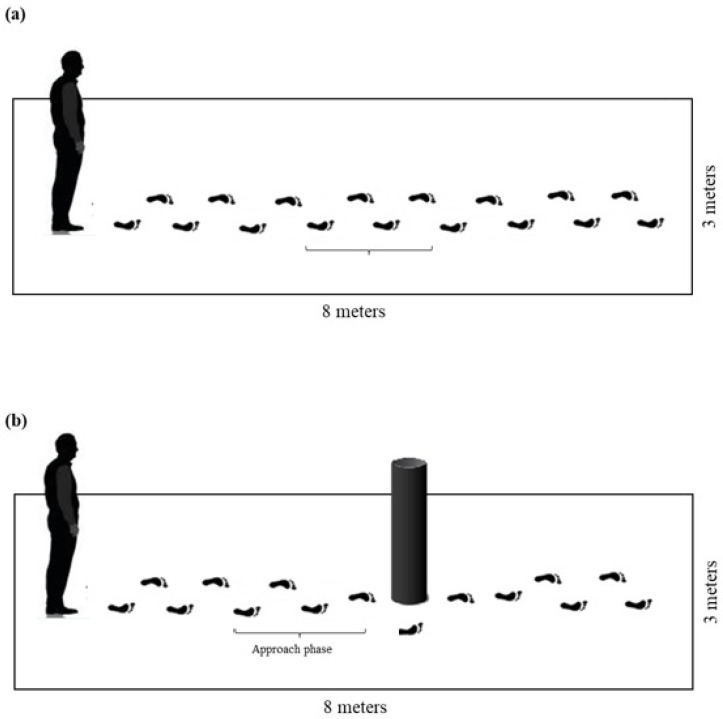
Schematic representation of the steps analyzed during (**a**) the unobstructed walking condition–the three central steps were analyzed; and (**b**) the obstacle circumvention condition–the three steps immediately preceding the obstacle (approach phase) were analyzed.

Eight Vicon Bonita cameras (100 Hz) were used to acquire the spatiotemporal parameters of gait (Vicon Motion System Ltd., Yarnton, Oxfordshire, UK). An image projector (Dell^®^) was also used (Dell Technologies, Austin, TX, USA) to simulate daily street-crossing situations, along with a pedestrian traffic light simulator. Thirty-nine passive reflective markers were placed on anatomical landmarks of the participants’ skin according to the Plug-in Gait Full Body model (Vicon^®^), and five markers were attached to the obstacle. Two evaluators were trained prior to the study to identify the anatomical landmarks. Following the recommendations of the Plug-in Gait Full Body model provided in the Vicon manual, the 39 markers were then properly positioned. Placement instructions can be found at: https://help.vicon.com/space/Nexus216/11607226/Full+body+modeling+with+Plug-in+Gait (accessed on 1 October 2020). Each step was defined as the interval between the initial contact of the heel with the ground and the subsequent heel contact of the opposite foot. In conditions without deviation, the three central steps were analyzed (Figure 3a). In the obstacle circumvention task, analysis focused on the approach phase, i.e., the three steps preceding obstacle circumvention (Figure 3b). Thus, a minimum of three steps per trial were analyzed for each condition and participant. When signal loss occurred, the images could be manually reconstructed within the software. However, if signal loss prevented avatar reconstruction, the participant was excluded from the analysis.

The three-dimensional coordinates of the participants’ and obstacle markers were exported in “.txt” format from the Nexus software 2.7 version (Vicon^®^) for further analysis in MATLAB version 2017a (MathWorks Inc., Natick, MA, USA). The spatiotemporal data were filtered using a fifth-order low-pass Butterworth filter with a cutoff frequency of 6 Hz. Subsequently, the following spatiotemporal gait variables were analyzed under all conditions: step length, step width, step duration, step speed, and percentage of double support [31,32].

### 2.5. Statistical Analysis

The SPSS^®^ software (PASW Statistics 18) was used to make statistical analysis. The data from the UPDRS, H&Y, and time-of-diagnosis scales are reported as mean and standard deviation for PG. We applied the *t*-test for independent samples to the data for sample characterization. After verifying the data’s normality and homogeneity, we adopted ANOVA for repeated measures, followed by the Bonferroni post-hoc test with two factors (groups and conditions). The level of significance was set at *p* < 0.05.

## 3. Results

### 3.1. Clinical Parameters

Table 1 presents the personal data, anthropometric measurements, and clinical assessments of the participants. Age, body mass, height, and MMSE averages were similar for both groups. Although BMI values differed significantly from CG and PG, the two groups were considered similar according to the Ministry of Health (2004), as the BMI classification of the older population considers the value > 27 kg/m^2^ as overweight. Regarding the history of falls, there were also no significant differences between groups.

### 3.2. Spatiotemporal Parameters of All Gait Conditions

**Figure 4:**
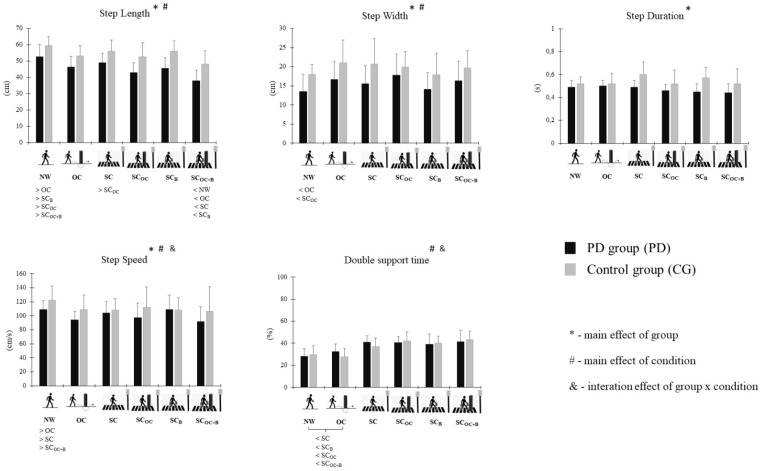
Main findings of each variable according to gait conditions in individuals with PD and CG. Bars indicate mean values, while error bars indicate standard deviation; *: indicates main effects of the group; #: indicates main effects of condition; &: indicates interaction effects of group × condition. > or <: indicates a higher or smaller value, respectively, compared to another condition. For all variables that showed a condition effect, the condition in which the post-hoc test showed statistically significant differences is indicated below.

The ANOVA main effects and interaction effects of spatiotemporal parameters are presented in Table 2. In the paragraphs below, we indicate the effects we found for each of those parameters (Figure 4).

CG subjects had greater values for all parameters compared to PG (step length, step width, step duration, and step speed–*p* < 0.05), except for double support time, which did not differ significantly from group to group.

Moreover, we noted a main effect of condition for all variables (*p* < 0.01) except for step duration. The values of step length during SC_OC+B_ were smaller in comparison to the other conditions (NW, SC, and SC_B_ [*p* < 0.001]; OC [*p* = 0.004]), except for SC_OC_, which did not show a statistically significant difference. In NW, the step length values were greater than in the SC_B_ (*p* < 0.02), OC (*p* < 0.001), SC_OC_ (*p* = 0.001), and SC_OC+B_ (*p* < 0.001) conditions. Last, step length was greater in SC than in SC_OC_ (*p* = 0.017). Step width during OC (*p* = 0.023) and SC_OC_ (*p* = 0.004) was greater than during NW. Step speed was faster during NW than in SC (*p* = 0.017), OC (*p* = 0.001), and SC_OC+B_ (*p* = 0.048). Finally, double support time was shorter during NW and OC in comparison to SC (*p* < 0.001; *p* = 0.001), SC_B_ (*p* < 0.001; *p* = 0.002), SC_OC_ (*p* < 0.001; *p* < 0.001), and SC_OC+B_ (*p* < 0.001; *p* = 0.001).

An interaction effect of group*condition was also found for step velocity and double support time. Step velocity was faster in CG than in PG during NW, OC, and SC_OC_ (12.19%, 15.41%, and 14.69%, respectively). Moreover, the step velocity of PG was 15.41% and 18.44% faster in the NW condition compared to OC and SC_OC+B_, while the step velocity of CG was 13% faster during NW in comparison to SC_B_. Finally, for PG, the double support time was shorter in NW than in SC (44.79%), SC_B_ (38.55%), SC_OC_ (43.48%), and SC_OC+B_ (46.95%), and in OC compared to SC (26.50%) and SC_OC_ (25.36%). Also, the values of these two conditions were shorter for CG: NW < SC (35.67%), SC_OC_ (43.04%), SC_OC+B_ (46.52%), OC < SC (34.43%), SC_B_ (45.45%), SC_OC_ (53.35%), and SC_OC+B_ (57.09%).

## 4. Discussion

Despite the growing interest in gait analysis in people with Parkinson’s disease (PD), most studies have focused on isolated tasks, typically evaluating basic locomotor patterns or street-crossing simulations under controlled conditions, without accounting for the simultaneous execution of everyday motor tasks. There is a notable lack of investigations that integrate multiple functional demands, such as obstacle circumvention and object carrying, during street crossing, a frequent and high-risk situation in the daily lives of individuals with PD. This gap is particularly relevant since individuals with PD exhibit deficits in divided attention, motor planning, and environmental adaptability, factors that may be further challenged in dual-task motor contexts. Therefore, studies that more ecologically simulate the demands of urban environments are needed to better understand the risks involved and to inform the development of targeted preventive and rehabilitative strategies.

In light of these considerations, our study was designed to address this gap by providing a more ecologically valid simulation of urban walking challenges experienced by individuals with PD. The aim of this study was to evaluate the spatiotemporal parameters of gait during daily tasks in individuals with PD, specifically during a street-crossing simulation performed concomitantly with obstacle circumvention and the execution of a dual motor task (carrying bags). The results confirmed both initial hypotheses: (i) individuals with PD showed smaller, narrower, slower, and shorter steps compared to neurologically healthy older adults; (ii) the complexity of the different gait conditions modified the gait spatiotemporal parameters of all individuals, except for the step-duration parameter. This is an interesting finding, since under the street-crossing conditions, the time allowed to complete the walking task was limited (8 s). Therefore, the expectation was that step duration would decrease to complete the tasks within that timeframe. In the following paragraphs, we will explain the differences found between the groups, as well as between conditions.

The worse performance of individuals with PD in the gait spatiotemporal parameters (step length, width, duration, and speed) compared to neurologically healthy older adults demonstrates the pathophysiology of PD. A decrease in dopamine production in the pars compacta of the substantia nigra, responsible for the impaired functioning of the basal ganglia, can lead to gait changes in this population [33,34]. Moreover, in PD, there is a change in motor behavior that originates from the failure of the task-planning area in processing information, resulting in the need for more time to react to a stimulus, especially when performing complex tasks [35]. Other possible factors that contribute to the shorter step length, speed, duration, and width we found in PG are bradykinesia, difficulty in initiating movements, and cholinergic dysfunction [36], which, according to Ford et al. [14], causes inattention, falls, delirium, and neural inhibition that increases the risk of having an accident while crossing the street [14]. Therefore, this study suggests that the changes in gait parameters may be directly related to this cholinergic dysfunction present in PD, which would be intensified in the presence of a dual task.

In individuals with PD, there is also impairment of executive function in the frontal lobe responsible for planning, sequencing, and performing motor and cognitive tasks, which, associated with attention control deficit, can interfere with the proper performance of gait [36,37,38,39,40]. According to Ford et al. [14], crossing the street involves executing several tasks at the same time, requiring the division of attention for each of them. This would then lead to motor impairment in PD patients since they have difficulty dividing their attentional focus. Moreover, deficits in visuospatial perception contribute to a greater risk when crossing the street in individuals with PD, and this risk is related to the motor severity of the disease [37]. The National Traffic Council (CONTRAN in Portuguese) [41] adopts a speed of approximately 120 cm/s for the complete crossing of roads. Based on this information, along with the results of this study, we noticed that the walking speed was only reached by the MN of the individuals in the CG. This shows that both the elderly population and both healthy and those with Parkinson’s disease may suffer a greater risk of having accidents in complex tasks, since this speed was not reached under other walking conditions.

This study corroborates that of Amaral–Felipe et al. [15] regarding the parameters of step length and speed, but on the other hand, in the referred article, the PG had a longer double support time than CG, unlike our study, in which there was no significant difference. Likewise, Monteiro et al. [42] also showed, through a narrative review, that the time of double support in individuals with PD is longer due to the search for postural restabilization and greater activation of postural and stabilizing muscles.

In the present study, the flexed posture of individuals with PD may have influenced the double support time, which reduces the fluidity of movements, as a consequence of the presence of compensatory movements of the hip, knee, and ankle. The flexed posture reduces the propulsion of the foot and makes the step flatter in order to maintain balance [43,44].

According to Williams and Martin [45], the percentage of double support increases as gait speed decreases due to the need to find strategies that increase gait stability in an attempt to make it safer. Such a mechanism is unconscious and requires an increase in hip, knee, and ankle range to occur in addition to an increase in ground reaction force and an increase in energy expenditure. Thus, the present study corroborates the results of Williams and Martin [45], since the double support increased in both groups under the most challenging gait conditions, such as street crossing, for example. Moreover, step speed was reduced significantly, suggesting a demand for adaptations to maintain gait control. On the other hand, although the PG adopted greater double support in order to better stabilize their gait, we know that these individuals have deficits such as alteration of the center of mass, reduction of trunk and hip dissociation, increase in energy expenditure, and decrease in range of motion, which would increase the risk of falling and having accidents in adverse environments [42].

Simieli et al. [31] compared strategies performed during obstacle avoidance between neurologically healthy elderly and people with PD. Both groups had difficulties in reducing speed and step length, increasing double support time and body instability, but the mechanism of the fit between the groups was different. While the CG was adjusted only in the final step of the detour, the PG depended more on the visual information of the obstacle throughout the task, thus increasing the attentional demand during the detour of the obstacle along the street crossing, projection of cars, and the headlight. There was a cortical overload in both groups, with more damage in the PG. This information suggests that the more complex the crossing environment is, the greater the risk of occurring accidents.

According to Oxley et al. [46], the increased complexity of the task impaired the performance of elderly people in complex traffic situations, such as crossing a two-way street with low vision, whereas street-crossing safety had better performance on one-way roads. Thus, our study corroborates the findings of Oxley et al. [46], as in more complex situations in both groups had worse gait performance due to a decrease in perceptive, cognitive, and physical skills acquired in non-ideal crossing decisions and behaviors, in which they are more impaired in groups with more difficulty [7].

Compared to the CG, a worsening of the gait performance of the PG was already expected, but under the condition of crossing the street carrying a bag, both groups practically equaled their step speed. This result can be attributed to the possible reduction in postural oscillation of PG individuals during loaded walking. This assumption is supported by the findings of Hill et al. [47], who observed a decrease in postural sway that results from carrying a light external load in advanced age groups. In their study, Hill et al. [47] did not identify a decrease in postural sway in younger age groups since, as the authors pointed out, changes in postural sway related to the act of holding bags seem to have a floor effect. Therefore, when postural oscillation is small and reaches a level that is close to the “physiological minimum”, there is no room for improvement, whereas in more advanced age groups, there may still be room for improvement in postural oscillation. Considering that individuals with Parkinson’s disease exhibit postural sway even in the early stages of the disease [48], it is possible that walking while carrying bags has benefited individuals with PD more.

The large prevalence of traffic accidents is not only a social issue, but also an important financial factor for the country. According to IPEA (Institute for Applied and Economic Research) [49], between 2007 and 2016, the number of deaths in traffic accidents in Brazil involving the elderly population, over 60 years, was about 66,000, in addition to costs related to traffic accidents in urban areas of approximately BRL 5.3 billion per year [49]. Therefore, to promote the inclusion and safety of elderly individuals and people with PD, as well as other degenerative conditions, it is essential to implement public policies that adapt traffic light timings to their specific needs. It is worth noting that crossing the street often involves concurrent tasks, such as those observed in our study, such as carrying bags and avoiding obstacles/pedestrians. Thus, providing longer intervals for crossing the street can facilitate the mobility of these vulnerable groups, ensuring they have sufficient time to cross safely. Investing in these adaptations not only promotes urban accessibility but also demonstrates a commitment to equity and the well-being of citizens. With this, this study aimed to support changes in traffic policies, welcoming the needs of the elderly and neurologically affected population, in addition to suggesting that complex tasks should be trained in a safe clinical environment associated with regular physical activity and dopaminergic drug interventions.

The differential aspect of this study was the analysis and comparison of different crossing-the-street conditions associated or not with obstacle avoidance and dual motor tasks. Other studies have analyzed crossing the street under a gait condition or even under a gait condition with different rhythms, but not with the performance of concomitant daily activities. Added to this, our method was stronger. We used equipment considered to be the gold standard for gait analysis (Vicon Motion System^®^) for the acquisition of kinematic data, which imposes greater reliability on our study in relation to the data, the solid number of repetitions in each condition (five trials per condition), which provides confidence in the data, as well as the age-matching with neurologically healthy participants.

However, this study has some limitations, such as the fact that data collection was conducted in a laboratory environment, which may have promoted a sense of security during street crossing, with no tests applied to assess the individuals’ visuospatial processing, and with the absence of a control group of young individuals. Moreover, while our protocol aimed to simulate realistic urban scenarios, it is important to acknowledge that controlled laboratory conditions may limit the external validity of the findings. Unpredictable environmental factors, such as weather variations, interactions with other pedestrians, and complex sensory stimuli, are not fully replicated in the laboratory. Additionally, there was a difference in gender distribution between groups, with a predominance of female participants in the control group and male participants in the Parkinson’s group. Although the primary outcomes are predominantly influenced by the presence of Parkinson’s disease, and the influence of gender on the analyzed gait variables is considered minimal, this imbalance may have introduced a potential source of bias. Therefore, our results should be interpreted considering these limitations, and future studies in real-world contexts are needed to confirm the applicability of our findings to daily life in people with Parkinson’s disease.

## 5. Conclusions

This study demonstrated that individuals with PD took smaller, narrower, slower, and shorter steps compared to neurologically healthy older people and that there was a change in the spatiotemporal gait parameters of all individuals, except for the step-duration parameter under the most difficult crossing conditions. Additionally, we concluded that both neurologically healthy older adults and those with PD may be at greater risk of having accidents while performing concomitant tasks such as dodging a person and holding a bag.

Based on our findings demonstrating impaired gait performance under complex dual-task conditions, our results have relevant clinical implications. Since individuals with Parkinson’s disease are more vulnerable to accidents in real-world environments that demand divided attention, our results highlight the need for targeted rehabilitation programs. These programs should include dual-task training in simulated environments and tailored education strategies for safe mobility in complex urban settings. In addition, our study may inform urban planning decisions, such as adapting pedestrian light durations or redesigning sidewalks and crossings, to enhance safety and autonomy for this population.

## Figures and Tables

**Table 1:** Anthropometric data, values of the Mini Mental State Examination, and level of involvement of the GP.

	CG (n = 18)	PG (n = 18)	*p* Values
Sex (M/F)	3 M/15 F	12 M/6 F	-
Age (years)	67.05 ± 6.46	68.72 ± 8.04	0.49
Body Mass (kg)	75.66 ± 17.15	65.94 ± 17.37	0.11
Height (m)	1.56 ± 0.07	1.60 ± 0.10	0.14
BMI (kg/m^2^)	30.95 ± 5.75	25.96 ± 5.20	0.01 *
MMSE (points)	27.39 ± 2.40	27.56 ± 2.20	0.82
UPDRS (points)	-	26.50 ± 10.62	-
H&Y	-	2.31 ± 0.48	-
Diagnostic Time (years)	-	5.91 ± 3.78	-
Number of falls (last year)	0.38 ± 0.84	0.61 ± 0.97	0.47

Values are presented by mean ± standard deviation. CG: control group; PG: Parkinson’s disease group; M: male; F: female; BMI: body mass index; MMSE: Mini Mental State Examination; UPDRS: Unified Parkinson’s Disease Rating Scale; H&Y: Modified Hoehn and Yahr Scale; *: indicates statistically significant effects.

**Table 2:** Spatial-temporal parameters values, ANOVA main effects and interaction effects for all conditions of gait in both groups.

Variables	Conditions	Groups		Main Effects (F(1,17) and *p* Values)		
		CG (n = 18)	PG (n = 18)	Group	Conditions	Group × Conditions
Step length (cm)	NW	59.36 (5.70)	52.61 (7.73)	F = 63.668 *p* ≤ 0.001	F = 22.943 *p* ≤ 0.001	F = 0.485 *p* = 0.781
	SC	56.03 (6.76)	49.00 (5.65)			
	SC_B_	56.01 (6.43)	45.61 (6.62)			
	OC	53.2 (6.20)	46.21 (6.77)			
	SC_OC_	52.59 (8.66)	42.83 (6.03)			
	SC_OC+B_	48.18 (8.18)	37.86 (6.58)			
Step width (cm)	NW	18.02 (2.61)	13.54 (4.47)	F = 11.776 *p* = 0.003	F = 5.858 *p* = 0.005	F = 0.854 *p* = 0.537
	SC	20.66 (6.66)	15.57 (4.73)			
	SC_B_	17.84 (5.65)	14.10 (4.38)			
	OC	21.02 (5.92)	16.69 (4.68)			
	SC_OC_	19.90 (4.09)	17.83 (5.49)			
	SC_OC+B_	19.64 (4.52)	16.37 (5.10)			
Step duration (s)	NW	0.52 (0.06)	0.49 (0.06)	F = 20.362 *p* ≤ 0.001	F = 1.839 *p* = 0.174	F = 2.945 *p* = 0.054
	SC	0.60 (0.11)	0.49 (0.06)			
	SC_B_	0.57 (0.09)	0.45 (0.07)			
	OC	0.52 (0.09)	0.50 (0.05)			
	SC_OC_	0.52 (0.12)	0.46 (0.05)			
	SC_OC+B_	0.52 (0.13)	0.44 (0.08)			
Step speed (cm/s)	NW	122.09 (20.53)	108.82 (12.59)	F = 7.624 *p* = 0.013	F = 5.921 *p* = 0.005	F = 3.819 *p* = 0.024
	SC	108.29 (16.45)	104.17 (16.49)			
	SC_B_	108.04 (18.12)	108.99 (20.54)			
	OC	108.82 (20.74)	94.29 (12.11)			
	SC_OC_	111.79 (29.46)	97.47 (20.45)			
	SC_OC+B_	106.51 (34.48)	91.88 (20.89)			
Double support time (%)	NW	29.58 (8.26)	28.22 (6.86)	F = 0.130 *p* = 0.723	F = 21.014 *p* ≤ 0.001	F = 3.267 *p* = 0.040
	SC	37.09 (7.86)	40.86 (5.95)			
	SC_B_	40.13 (6.11)	39.10 (9.12)			
	OC	27.59 (7.66)	32.30 (6.99)			
	SC_OC_	42.31 (8.00)	40.49 (5.42)			
	SC_OC+B_	43.34 (7.83)	41.47 (10.45)			

Values are presented by mean (standard deviation). The *p*-values in bold indicate statistically significant differences. CG: control group; GP: Parkinson’s disease group; NW: normal walking without crossing the street and or performing any concomitant task; SC: walking with a street crossing, in which the volunteers were instructed to walk without performing any other concomitant task; SC_B_: walking with street crossing carrying bags with weight, in which the volunteers were instructed to walk and carry loads corresponding to 10% of body weight in bags similar to shopping bags, equally divided between the two upper limbs; OC: walking without crossing the street with obstacle circumvention; SC_OC_: walking with street crossing and circumventing an obstacle; SC_OC+B_: gait with street crossing carrying bags and circumventing the obstacle.

## Data Availability

No new data were created or analyzed in this study.
